# Food Types in the Diet and the Nutrient Intake of Obese and Non−Obese Children

**DOI:** 10.4008/jcrpe.v1i1.5

**Published:** 2008-08-04

**Authors:** Muazzez Garipağaoğlu, Yusuf Sahip, Nurten Budak, Öznur Akdikmen, Tuğçe Altan, Melis Baban

**Affiliations:** 1 Istanbul University, Istanbul Faculty of Medicine, Department of Pediatrics, Istanbul, Turkey; 2 Istanbul University, Institute of Child Health, Family Health Department, Istanbul, Turkey; 3 Erciyes University, Department of Nutrition and Dietetics, Kayseri, Turkey; +90−212−631 17 10+90−212−631 17 10sahip@istanbul.edu.trIstanbul Üniversitesi, Çocuk Sağlığı Enstitüsü, 34093 – Çapa, İstanbul / Turkey

**Keywords:** Obese, non−obese children, nutrient intake, food variety, recommendations

## Abstract

**Background**: Childhood obesity has reached epidemic proportions world−wide.

**Objective**: To compare the types of food in the diet and the nutrient intake of obese children with those of non−obese children.

**Methods**: A total of 95 obese and 592 non−obese children aged between 6 and 10 years participated in the study. A body mass index (BMI) value exceeding the 95th percentile for age and gender was taken as the criterion for obesity. Three−day food consumption was recorded and evaluated according to standard international recommendations.

**Results**: Macronutrient intake was adequate in both obese and non−obese children. Energy intake of the obese children was significantly higher than that of the non−obese children. Micronutrient intake except fiber of both groups, calcium intake of obese children and vitamin A intake of non−obese children were higher than recommended amounts. The obese children consumed excessive fat and sugar, but less fruit and vegetables as compared to the non−obese children, and less than the recommendations of the food guide pyramid as adopted by the US Department of Food and Agriculture and the Department of Health and Human Services.

**Conclusion**: The implementation of educational programs on nutrition may be important for promoting knowledge about healthy eating among obese children.

**Conflict of interest:**None declared.

## INTRODUCTION

Childhood obesity has reached epidemic proportions. In a 2004 report of the World Health Organization (WHO) it was stated that 10% of children were estimated to be overweight or obese world−wide.(1) Wang and Lobstein(2) have recently reported an increase in prevalence of overweight and obesity among children in more than sixty countries from different regions of the world. It has been estimated that over 46% of school−age children will be overweight in the United States by the year 2010. A similar trend has been observed in Turkey.(3, 4)

Obesity has negative implications for the physical and emotional health of children and adolescents. Cardiovascular disease, type 2 diabetes mellitus, degenerative joint disease and depression in later life are some of the undesirable consequences of obesity in children.(5, 6) Almost 60% of overweight children aged between 5 and 10 years are found to have at least one risk factor for future cardiovascular disease such as high blood pressure, abnormal lipid profile, elevated insulin level and more than 25% two or more of these risk factors.(5) Furthermore, evidence from experimental and longitudinal cohort studies have shown that overweight children are more likely to suffer from psychological problems.(7) Prevention and treatment of obesity is a public health priority world−wide.

Overweight and obesity are influenced by many factors including hereditary tendencies, environmental and behavioural factors.(8) It is difficult to determine which one has the strongest effect on obesity. However, recent evidence suggests that dietary factors and physical activity patterns are strongly associated with increasing body weight.(9, 10, 11, 12, 13, 14) The association between obesity and decreasing physical activity was assessed in several studies.(15, 16, 17) Not only is the role of diet in development of obesity poorly understood, but also the limitations of dietary methodology make it difficult to show an association between diet composition and prevalence of obesity.(18, 19) There is an urgent need to identify dietary factors that contribute to obesity among children and adolescents so that preventive efforts can be effectively targeted at an early age. This present study aimed to compare food varieties in the diet and the nutrient intake of obese and non−obese children.

## MATERIALS AND METHODS

**Subjects**: The study was carried out in a public primary school in an urban district. The data were collected between November and December 2006.

Obesity prevalence usually changes from 10% to 15% among school−aged children.(2, 4) Assuming this proportion to be 15%, and allowing for a confidence level of 95% and a margin of 0.03 for errors, it was calculated that the sample size required for the study was a total of 545 children between 6−10 years of age. Many obese children come for treatment to our institution from urban districts of medium socioeconomical level which include a total of 21 schools. Fourteen of these 21 schools were found to have at least 545 students aged between 6−10 years. The study school was selected randomly from among these 14 schools using a computer−generated randomization program. The school had classes from 1st to 5th grades, and each grade had 4 sections, with 35−40 students. In total the school had 20 classes and 721 students. Fourteen children with acute and chronic illness were excluded from the study, and 20 students refused to partake in the study. Thus the study was conducted on a group of 687 children.

Weight and height were measured by an experienced dietitian. Weight was measured by a portable scale with an accuracy of 0.1 kg, with the children in their underwear. Height without shoes was measured using a portable measuring device to an accuracy of±0.5 cm which was fixed on the wall, with the child standing straight with his/her back, buttock, heels and head against the wall. Body mass index (BMI) (kg/m^2^) and BMI SDS (standard deviation scores) of children were calculated by using the standard BMI score developed for Turkish children.(20) A BMI value exceeding the 95^th^ percentile for age and gender was taken as the criterion for obesity. The children were grouped as obese and non−obese.

Data relating to characteristics of parents and children were obtained by a structured questionnaire given to parents, which included questions on education and employment status of the parents, birth weight and age of onset of obesity in the child. The study was approved by ethical committee and informed consent was taken.

**Collection and assessment of food intake**: The dietary section of the study conducted over three−consecutive days (one day during the weekend) consisted of food consumption records based on household measures of quantities, e.g. the serving size being designated as portion, cup, mug, small or big glass, teaspoon, tablespoon, as explained to the families, who were requested to report the food intake of their children on this basis. The data on the diets were coded and recorded by a dietitian and analyzed by a biostatistician. The dietary records of the children were analyzed for total energy, macro and micronutrient content using the Bebis software dietary program developed for the national nutrition programme.(21) Mean energy and nutrient intake of the children were evaluated according to the recommended dietary allowances (RDAs) for Turkey.(22)

**Food group consumption**: All foods and beverages consumed by the children were categorized into groups based on methods developed by the US Department of Agriculture for disaggregating mixtures into their ingredients and assigning ingredients into groups in a Pyramid as seen in Figure 1. For example, if a vegetable was eaten with meat, the meat contributed to the number of servings of meat, the vegetable contributed to the servings of vegetables, and so forth. Foods not consumed as mixtures were assigned directly to their appropriate group in the Pyramid.

Weights for each food were converted as grams into the number of food group servings based on the methods proposed by Cleveland et al.(23) A serving size was defined according to the Pyramid as follows: fruit, one medium piece of fruit; vegetable: half cup/two tablespoons of cooked vegetable, 1 cup raw leafy vegetable, one medium piece of raw vegetable; cereal: one slice of bread, two tablespoons of cooked rice, cracked wheat or pasta, one slice of cake and pastry that contains an amount equivalent to that in a standard slice of bread (25 g); dairy: one glass of milk, one cup of yogurt, 1 oz. of cheese and two glasses of ‘ayran’ (drink made of yoghurt and water). Fat was defined as the ‘fat added’ to the food when cooking or ‘fat added’ at the table in the form of spreads, sauces, and salad dressings. Fat which occurs naturally in foods, such as milk, eggs, meat were not counted as added fat. Sugars were also defined as the ‘sugar added’ to foods such as cakes, beverages, jams and ice cream as well as the sugars consumed separately. Sugar which occurs naturally in food, such as milk and fruit were not counted as added sugars.

**Data analyses and statistics**: All calculations were performed by using Statistical Package for Social Science (SPSS) version 11.9 (customer ID: 115429). Data on the characteristics of parents and children were presented as mean±standard deviation (SD) and percentage (%). Energy and nutrient intakes of children were given as mean±SD. Student t−test was used to compare the means. Mann Whitney U test was used to compare the data of obese and non−obese children. P value <0.05 was considered as statistically significant.

**Figure 1:**
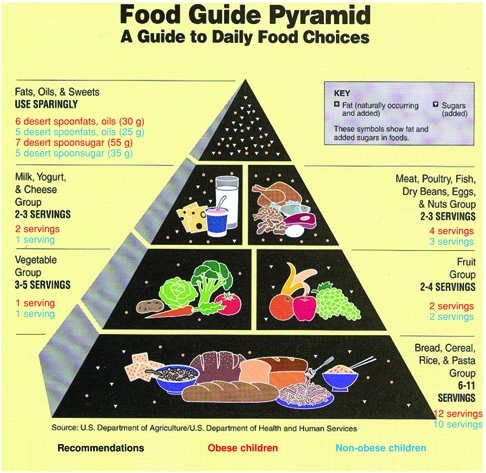
The pyramid for the five major food groups. The recommended amounts for school−age children are listed in black letters, while our findings for the obese group are written in red and those for the non−obese group in blue characters.

## RESULTS

In this study, we evaluated the variety of food, energy and nutrient intakes of 95 (13.8%) obese and 592 (86.2%) non−obese children aged between 6 and 10 years. The demographic characteristics of the parents and children are summarized in Table 1. Chronological ages and birth weights of the obese and the non−obese children were comparable. Age of onset on obesity among children was 3.9±2.3 years. Mean BMI and BMI SDS of the obese and the non−obese children were 26.1±3.1 kg/m^2^, 2.4±0.6 and 16.7±2.0 kg/m^2^, −0.1±0.5 respectively. The children’s fathers had attended school for more years than the mothers. Most of the mothers of the obese and the non−obese children were housewives (54% and 56% respectively), while all fathers in the two groups held jobs as employees.

Daily energy and nutrient intake of the obese and the non−obese children and their evaluation according to RDAs are presented in Table 2. While the obese children met 105% of daily energy requirements, energy intake of the non−obese children was 98% of RDAs. Moreover, calcium (Ca) intake of the obese children and vitamin A intake of the non−obese children, and other nutrient intakes except fiber of both groups were over the recommendations. Energy and vitamin B_12_ intakes of the obese children were significantly higher than the non−obese, while vitamin C and Ca intakes of non−obese children were higher than the obese (p<0.01). The energy intake from fat, carbohydrate and protein of all children were 34.0%, 50.0% and 16.0% respectively and there were no significant differences between the energy intake from protein, carbohydrate and fat of the obese and the nonobese children.

Mean number of servings consumed from the five major food groups is presented in the context of a pyramid recommended for school−age children (Figure 1). Consumption of the obese children from the cereal, meat, fat and sugar groups of nutrients were higher than the recommended and also higher than that of the non−obese children. Although, vegetable−fruit and dairy group intakes of all children were below the recommended allowances, the obese children consumed less from these food groups as compared to the non−obese children. In addition, obese children consumed more snack foods, such as cake, pastry, cookies, ice−cream, fast−food and soft drinks, than the non−obese children.

**Figure 1:**
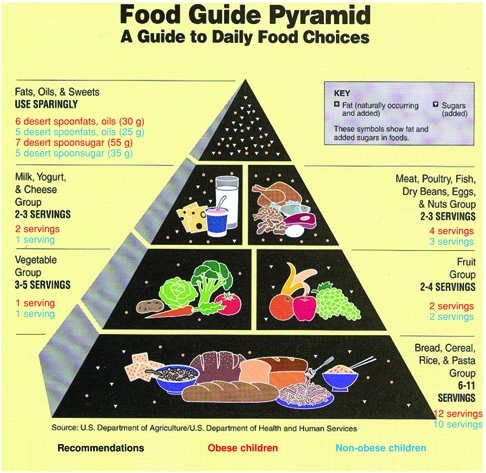
The pyramid for the five major food groups. The recommended amounts for school−age children are listed in black letters, while our findings for the obese group are written in red and those for the non−obese group in blue characters.

**Table 1:**
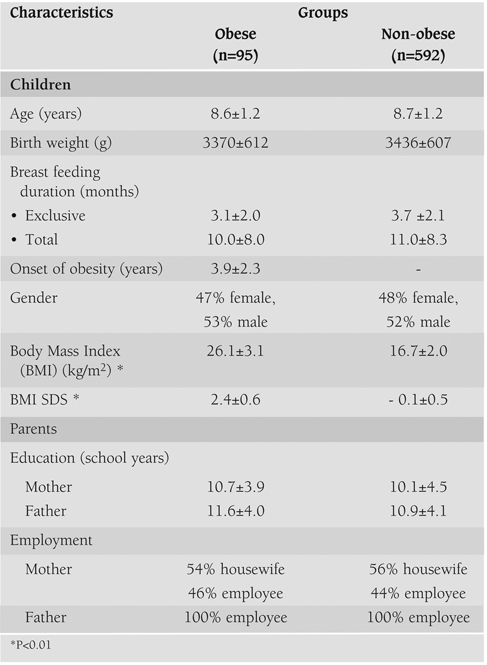
The characteristics of the children and their parents

**Table 2:**
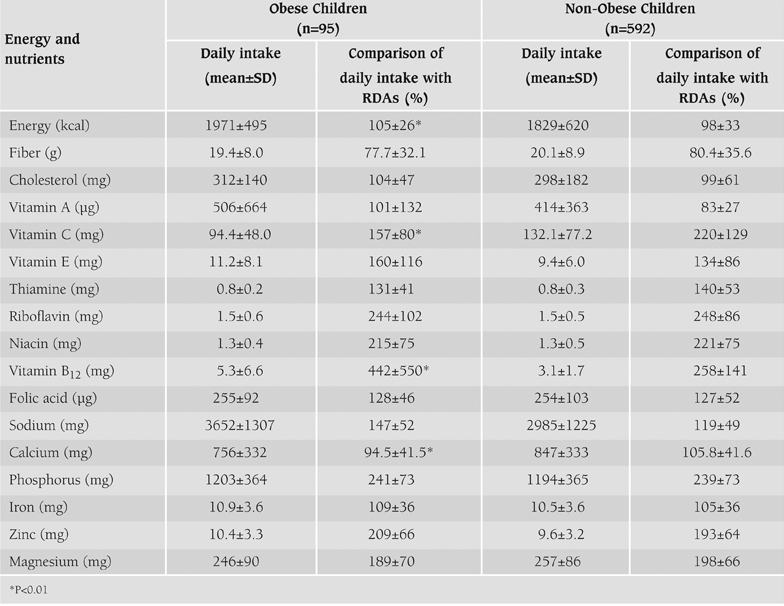
Mean daily energy and nutrient intake of obese and non−obese children and evaluation based on RDAs

## DISCUSSION

One of the most important findings of this study is that the energy intake of obese children is significantly higher than that of non−obese children. The other significant finding is that all children and especially the obese children consumed excessive fat and sugar but insufficient vegetable and fruits. Obesity is usually thought to result from excess energy intake and inadequate physical activity.(16) Huang et al.(24) reported that daily energy intake and energy density of the food consumed at meals were significantly and positively related to BMI. Similarly, we found that daily energy intake of obese children was 140 kcal more than those of non−obese children. In the Bogalusa heart study it has been reported that total energy intake of the 10 year−old children remained unchanged between 1973 and 1994, while energy intake per unit of body weight decreased from 65.5 kcal/kg in 1973 to 55.4 kcal/kg in 1994. In fact, this result was due to the increase in the weights of the children over the two decades.(25) Likewise, Troiano et al.(26) reported that mean energy intakes of children aged between 6 and 11 years decreased slightly or remained stable from the early 1970s to 1994. An increase of body weight noted when the energy intake decreases or remains the same, is explained by decreasing physical activity.(17, 18, 19, 26) We have estimated that if obese children do not spend energy equivalent to more than 5% of RDA’s through physical activity, their body weight will increase by about 4 kg in a year.

An elevated consumption of fats and sugars and a low consumption of fruits and vegetables among children has been reported from several countries.(9, 10, 27, 28, 29, 30, 31) In the last 20 to 30 years, it has been observed that children have consumed more food out of home, in restaurants and especially in fast−food serving places. They have come to consume much more junk food as well as a lot of fruit juice and soft drinks, and their diet includes more fat and sugar and less vegetable and fruit than the recommended. Also, the portions served to children are large. Thus, negative changes in eating habits have led to an increase in energy input and the resultant increase of obesity among children.(9, 11, 24, 32, 33, 34, 35, 36) A faulty nutritional pattern plays an important role in promoting the increase of adiposity in children.(16) Munoz et al.(29) indicated that only 1% of children are meeting recommendations for the groups of foods in the Pyramid and also demonstrated that the diet of children greatly exceeded the recommendations for fat and added sugars. Brady et al.(28) have also indicated that children consume excessive energy (46%) from the foods at the “tip” of the Pyramid. The acceptable energy percentage of intake from fats changes between 25 and 35% according to the Institute of Medicine, Food and Nutrition Board.(37) In accordance with Brady(28) and Munoz’s(29) reports, all children in our study were found to consume excessive amounts of fat and the children in the obese group were found to consume excessive sugar in particular. The percentage of energy intake from fat by our study groups was found to be at the upper limit.

In our study, excessive fat consumption in both groups is thought to result from excessive consumption of snacks such as cakes, pies, cookies and fast−food articles. Excessive sugar consumption in the obese group resulted from consumption of excessive amounts of ice cream, jam, patisserie and soft drinks compared to the non−obese children.

In contrast to the excessive intake of fats and sugar, the fruit and vegetable consumption by the children is generally insufficient. Numerous surveys have reported that consumption patterns are considerably below that based on the recommended amounts.(28, 29, 30, 31) Similarly, in our study it was also observed that the children consume insufficient amount of fruits and vegetables, the obese children consuming one portion of vegetable and two portions of fruit. (Figure 1). Excessive fat and sugar versus inadequate fruit and vegetable consumption has been linked not only to the promotion of obesity, but also to the increase in the risks of developing cardiovascular diseases and certain types of cancer.(36) Therefore, the children should be encouraged to replace cereal snacks such as cakes and pastry, with fruits, and to replace soft drinks with skimmed milk and yoghurt. Even such a slight dietary modification would decrease fat and sugar intakes among children, and contribute to prevention of obesity in childhood and consequent reduction in the incidence of chronic diseases in adulthood.

The most important limitation of this study was that we were unable to assess the impact of physical activity on obesity. In addition to diet, physical activity plays a role in the diet−obesity equation.(13, 14, 15, 16, 38) The study by Andersen et al.(39) has shown that children who watched 4 or more hours of television per day had higher body fat and BMI values than those who watched less than 2 hours per day. 

In conclusion, this study showed that obese children consume excessive fats and sugar but less fruits and vegetables than amounts recommended for these groups of foods in the food guide Pyramid. These results suggest that implementation of education programs on nutrition may be important for promoting knowledge about healthy eating among children. A remedy for promoting knowledge of healthy eating guidelines may include a strong family and school−based nutritional education program. We strongly believe that such programs which also include enhanced physical activities should be effective to reduce childhood obesity.

**Figure 1:**
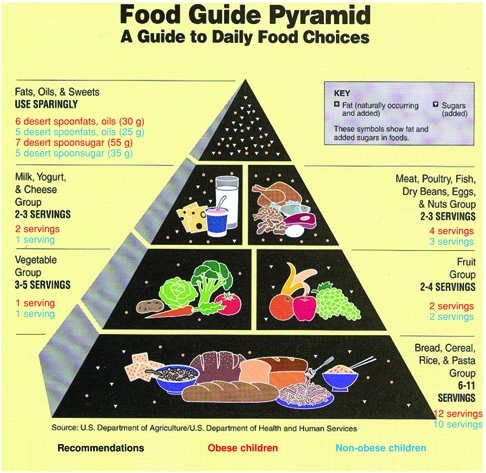
The pyramid for the five major food groups. The recommended amounts for school−age children are listed in black letters, while our findings for the obese group are written in red and those for the non−obese group in blue characters.

## ACKNOWLEDGEMENT

We would like to thank Associate Prof. Dr. Helaine K Minkas for language revision and to Biostatistician Sevda Özel for her help in the statistical evaluation of our study.
